# Polychlorinated biphenyls target Notch/Dll and VEGF R2 in the mouse placenta and human trophoblast cell lines for their anti-angiogenic effects

**DOI:** 10.1038/srep39885

**Published:** 2017-01-10

**Authors:** Satyan Kalkunte, Zheping Huang, Eliana Lippe, Sunil Kumar, Larry W. Robertson, Surendra Sharma

**Affiliations:** 1 Department of Pediatrics, Women and Infants Hospital of Rhode Island-Warren Alpert Medical School of Brown University, Providence, Rhode Island, USA; 2 Division of Reproductive and Cyto-Toxicology, National Institute of Occupational Health (Indian Council of Medical Research), Ahmedabad, Gujarat, India; 3 Department of Occupational & Environmental Health, College of Public Health, The University of Iowa, Iowa City, IA, USA

## Abstract

The intrauterine environment is particularly vulnerable to environmental exposures. We previously established a mouse model that provided evidence for pregnancy complications and placental anti-angiogenesis in response to Aroclor 1254 (A-1254), a mixture of polychlorinated biphenyls (PCBs). Importantly, these effects were observed in IL-10^−/−^, but not wild type, mice, suggesting that IL-10 deficiency predisposes to pregnancy disruptive effects of environmental toxicants. However, the mechanisms by which PCBs cause anti-angiogenic effects are unclear. Here, we evaluated PCB-mediated anti-angiogenic effects by diverse but complementary approaches, including HUVEC-mediated trophoblast invasion in nude mice, *in vitro* three-dimensional capillary tube formation involving HUVEC and/or HTR8 trophoblasts, and aortic ring endothelial cell outgrowth/sprouting. Taken together, our data suggest that PCBs act as potent anti-angiogenic agents. Importantly, we show that treatment of pregnant IL-10^−/−^ mice with A-1254 resulted in placental activation of the Notch/Delta-like ligand (Dll) pathway, a master regulator of cell-cell interaction and vascular patterning. Similar results were obtained with HUVEC and HTR8 trophoblasts. Rescue of A-1254-induced disruption of HUVEC-based tube formation by γ-secretase inhibitor L1790 confirmed the critical role of the Notch/Dll pathway. Our data suggest that PCBs impart pregnancy disruptive functions by activating the Notch/Dll pathway and by inducing anti-angiogenic effects at the maternal-fetal interface.

Polychlorinated biphenyls (PCBs) and their congeners have been widely characterized as ubiquitous environmental toxicants^1^,^2^,^3^,^4^. Extensive use of PCBs in various products and their unregulated disposal into environment for over five decades before their ban in1970’s has resulted in human and wildlife exposure. Moreover, unintentional by-products of organic pigments have continued to contribute to PCB exposure^5^. Due to their structural nature, these chlorinated hydrocarbons are highly stable and resistant to physical, chemical and biological degradation. Thus, PCBs have long half life and residues have been detected in milk, human adipose tissue, fish, building material, paint, pigment, wildlife and sediments, raising concerns over their potential toxicity^3^,^6^,^7^,^8^,^9^.

The toxic and inflammatory effects of PCBs have been observed both in humans and laboratory animals^10^,^11^,^12^,^13^,^14^,^15^,^16^,^17^. Epidemiologic studies suggest that human exposure to environmental toxicants including PCBs is associated with dermal, respiratory, and reproductive anomalies^18^. The fetus and infants are particularly vulnerable to chemical exposure due to the critical developmental periods^19^. Low birth weight in newborns, spontaneous abortion, and neurological problems including attention deficit disorder and autism are reported^20^,^21^,^22^,^23^,^24^,^25^. In support of these epidemiological findings, we established a mouse model that provided evidence for pregnancy complications in which genetic stress of IL-10 deficiency combined with exposure to Aroclor 1254 (A-1254), a mixture of more than 100 PCB congeners, resulted in preterm birth, fetal growth restriction, loss of righting reflex, and increase in amniotic fluid accumulation^11^. Intriguingly, we demonstrated that administration of PCBs during pregnancy in IL-10^−/−^ mice resulted in impaired transformation of blood vessels (spiral arteries) in the decidua and inhibition of the angiogenic water channel aquaporin 1 (AQP1) in uteroplacental tissue. IL-10 is a critical cytokine for normal pregnancy outcome. This cytokine entails potent anti-inflammatory and vascular functions^26^,^27^. We and others have demonstrated that IL-10^−/−^ mice are particularly sensitive to low doses of inflammatory triggers resulting in pregnancy complications^28^,^29^,^30^. Thus, the question arises whether PCBs activate vital anti-angiogenic pathways to cause vascular deficiency at the maternal-fetal interface and ensuing adverse pregnancy outcome.

Angiogenesis at the maternal-fetal interface is a result of cross-communication between different cell types abundant locally, including specialized natural killer cells (uNK), invading trophoblasts and endothelial cells^31^,^32^,^33^,^34^,^35^. In general, vascular endothelial growth factor (VEGF) is indispensable for the formation of primary vascular network and secondary angiogenesis^36^. VEGFs signal through their cognate receptors, of which the kinase activity of VEGF R2 is considered critical for trophoblast invasion^33^. The temporal and spatial function of VEGF in development and remodeling of the vascular system is regulated via “on” or “of” switching by endogenous inhibitors. In this context, the Notch receptors, their Delta-like ligands (Dll) and signaling pathways are considered vital for regulated vascular morphogenesis and remodeling^37^,^38^,^39^. Notch signaling plays an important role in cell-cell interaction and vascular patterning. The Notch family is represented by four Notch receptors- Notch1, Notch2, Notch3 and Notch4- and five ligands- Jagged1 and Jagged2- and Dll1, Dll3, and Dll4 ligands. Dll binding to Notch receptors induces the proteolytic release of Notch intracellular domain (NICD) which translocates to the nucleus to form transcriptional complexes and induces downstream expression of transcription factors such as HES and HEY^40^,^41^,^42^,^43^,^44^,^45^. Transcription factor Hey2 has been shown to inhibit VEGF R2 expression and angiogenesis^46^. Mouse embryonic vasculature has been shown to be influenced by signaling through Notch receptors, particularly Notch1 and Notch4^47^. Interestingly, Dll4 functions as an endogenous factor that negatively regulates the VEGF-mediated signaling^46^. Further, the importance of the Dll4 gene in normal vascular development has been demonstrated by gene knockout experiments in mice with embryonically lethal outcomes^48^. On the other-hand, over-expression of Dll4 is associated with severely compromised vasculature^49^.

We hypothesize that A-1254 perturbs the Notch/Dll-mediated angiogenic-antiangiogenic balance in the placenta that negatively influences pregnancy outcome. In this study, we demonstrate that PCBs (A-1254) act as anti-angiogenic agents and activate the Notch4/Dll4 pathway in the placenta of pregnant IL-10^−/−^ mice. These studies can form a basis for single congener specific evaluation of their angiogenic and anti-angiogenic functions in pregnancy.

## Results

### A-1254 treatment of HTR8 trophoblast cells impairs their invasive ability in an *in vivo* Matrigel plug assay

**Figure 1:**
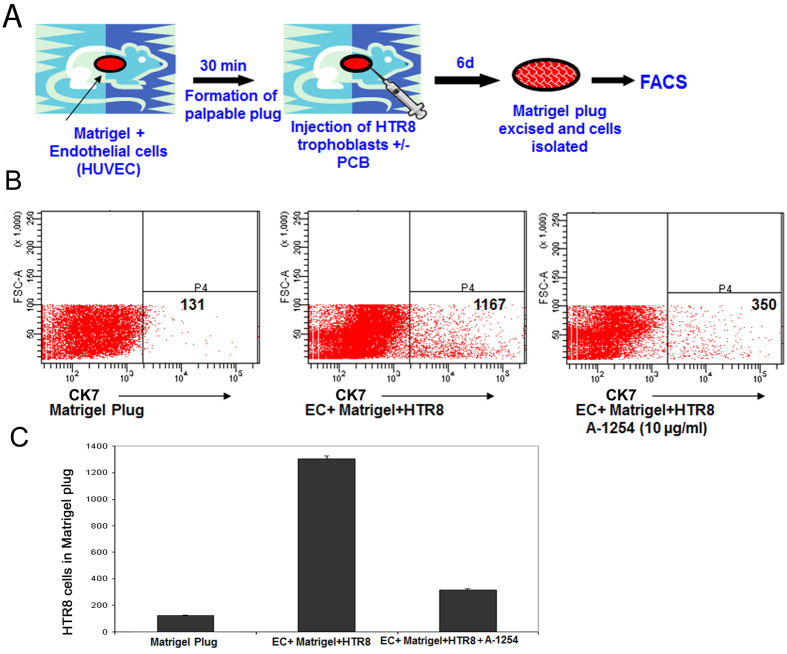
Effect of A-1254 on trophoblast invasion in an *in vivo* Matrigel plug assay. Panel A is the schematic presentation of the process for the *in vivo* Matrigel plug assay. Cytokeratin 7 (CK7)-positive human trophoblast HTR8 cells (7.5 × 10^5^) were treated with A-1254 (10 μg/ml) or vehicle (DMSO) for 24 hr, washed and injected in the periphery of the palpable Matrigel plug containing HUVECs as described in Methods. After 6 days, the cells were retrieved from the plug, stained for CK7 and analyzed by flow cytometry. Panel B shows the quantity of CK7-positive cells (HTR8 cells) from the Matrigel plug. In the left panel, no endothelial cells were used in the assay (negative control); in the middle panel, HTR8 cells were treated with vehicle; in the right panel, HTR8 cells were treated by with A-1254. Figure C shows quantification of FACS data from three experiments. The P value between the A-1254-treated and untreated samples was calculated to be <0.0001.

As described below, we employed diverse but complementary approaches to obtain direct evidence that PCBs induce anti-angiogenic functions. Endothelial cells exhibit chemoattracting ability for invasive trophoblast cells and this mimics physiological events associated with spiral artery transformation^31^,^32^. To study whether the trophoblast’s invasive ability is indeed impaired by treatment with A-1254, we evaluated its effect in an *in vivo* Matrigel plug assay. For this approach, we used human endothelial cells HUVECs and human first trimester extravillous trophoblast HTR8 cells (see Methods). Since PCBs show acute effects in IL-10^−/−^ mice, we used HTR8 trophoblasts as these cells are defective in IL-10 production^50^. As shown schematically in Fig. 1A, HTR8 cells when injected at the periphery of the Matrigel plug invade the plug in response to trophic signals from endothelial cells. The plug invading HTR8 cells can be identified by flow cytometry by staining for human cytokeratin 7 (CK7)-positive cells. As shown in Fig. 1B (right panel), pre-treatment of HTR8 cells with A-1254 for 24 hr resulted in poor presence of CK7-positive cells in the Matrigel plug as compared to vehicle treated group, suggesting that exposure to PCBs impaired the invasive ability of trophoblast cells. The decrease in HTR8 cells in PCB treated group was not due to cytotoxicity (data not shown). Matrigel plugs devoid of endothelial cells were used as negative controls and did not show significant presence of HTR8 cells (Fig. 1B, left panel). Data from three different experiments have been plotted in Fig. 1C to quantify the A-1254-mediated effect.

### A-1254 treatment inhibits endothelial cell outgrowth/sprouting from mouse aorta

**Figure 2:**
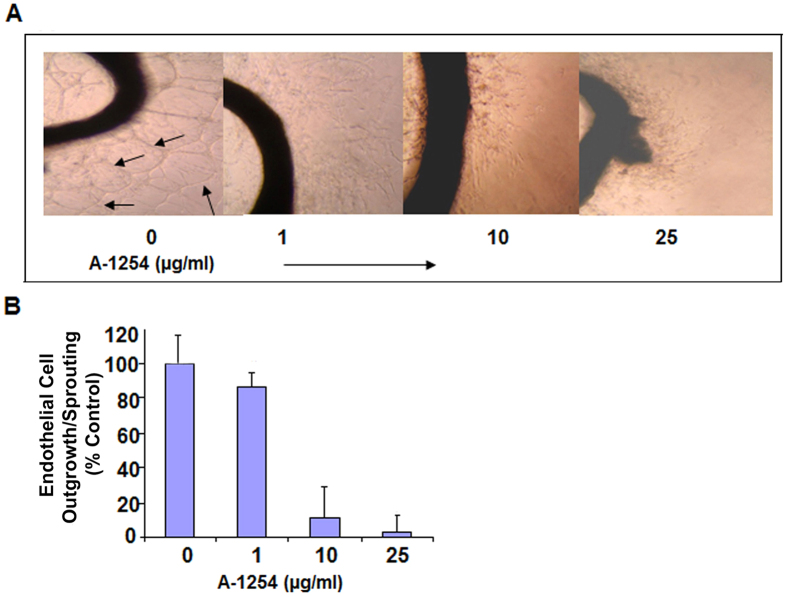
A-1254 inhibits endothelial cell outgrowth/sprouting from mouse aorta explants. Panel A shows aortic ring explants embedded in Matrigel, treated with A-1254 in a dose dependent manner, incubated for 6 days, and monitored for the endothelial cell outgrowth/sprouting (see arrows). Growth medium with or without A-1254 was changed every 48 hr. In vehicle-serum treated explants, endothelial cells characteristically sprout and form three-dimensional structures, and this process is inhibited by A-1254 in a dose-dependent manner. Panel B shows quantification of endothelial cell outgrowth/sprouting as determined by the MTS assay. The calculated P value signifying the inhibition of endothelial cell outgrowth/sprouting is <0.0001.

Using the aortic ring assay as described in Methods, we evaluated the effect of A-1254 on endothelial cell outgrowth and sprouting. A-1254 inhibited the endothelial cell vessel formation in mouse aorta explants in a dose dependent manner (Fig. 2A), demonstrating that PCB treatment is capable of modulating endothelial cell vascular activity. The extent of inhibition by A-1254 was quantified using the MTS assay and is expressed as percent of endothelial cell outgrowth/sprouting (Fig. 2B).

### A-1254 treatment of HTR8 trophoblast cells disrupts endovascular cross-talk with HUVECs and formation of three-dimensional capillary tube structures

**Figure 3:**
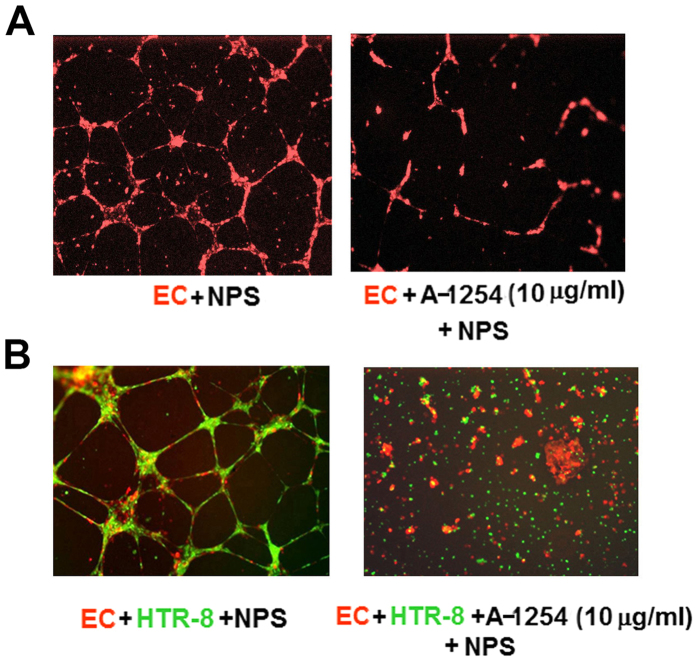
The effect of A-1254 on three-dimensional tube formation involving HUVECs and/or HTR8 trophoblast cells. HUVECs and HTR8 cells were labeled with red and green cell trackers, respectively. These cells were cultured overnight on Matrigel in the presence of normal pregnancy serum (NPS) supplemented with vehicle or A-1254 (10 μg/ml) as described in Methods. Panel A: HUVECs (red); Panel B: HUVECs (red) and HTR8 cells (green). These data represent at least three experiments.

As discussed earlier, invading trophoblast cells interact with endothelial cells in uterine spiral arteries to transform them into resistant-free capillary structures^31^,^32^. We developed an assay to mimic this process *in vitro* which depends on formation of three-dimensional tube-like capillary structure formation between HUVECs and first trimester trophoblast cells cultured on Matrigel^33^. Using this assay we evaluated whether treatment with A-1254 influenced HUVEC-mediated capillary structure formation or endovascular cross-talk between HUVECs and HTR8 cells. HUVECs (labeled red) were cultured or co-cultured with HTR8 cells (labeled green) on Matrigel in the presence of normal pregnancy serum (NPS) with and without A-1254 (10 μg/ml). As shown in Fig. 3A, HUVECs formed three-dimensional tube structures when cultured in the presence of NPS, whereas A-1254 treatment disrupted this process. Likewise, HUVECs and HTR8 cells formed three-dimensional capillary tube-like structures in the absence of PCB treatment, supporting our previous observations (Fig. 3B). However, inclusion of A-1254 in the assay profoundly disrupted this process (Fig. 3B), implying that PCB congeners present in A-1254 harbor potent anti-angiogenic activity. A-1254 at 10 μg/ml was not cytotoxic to either HUVECs or HTR8 cells (data not shown). These observations are in agreement with the data presented in Figs 1 and 2.

### Exposure to A-1254 up-regulates DLL4 and Notch 4 in the mouse placenta

**Figure 4:**
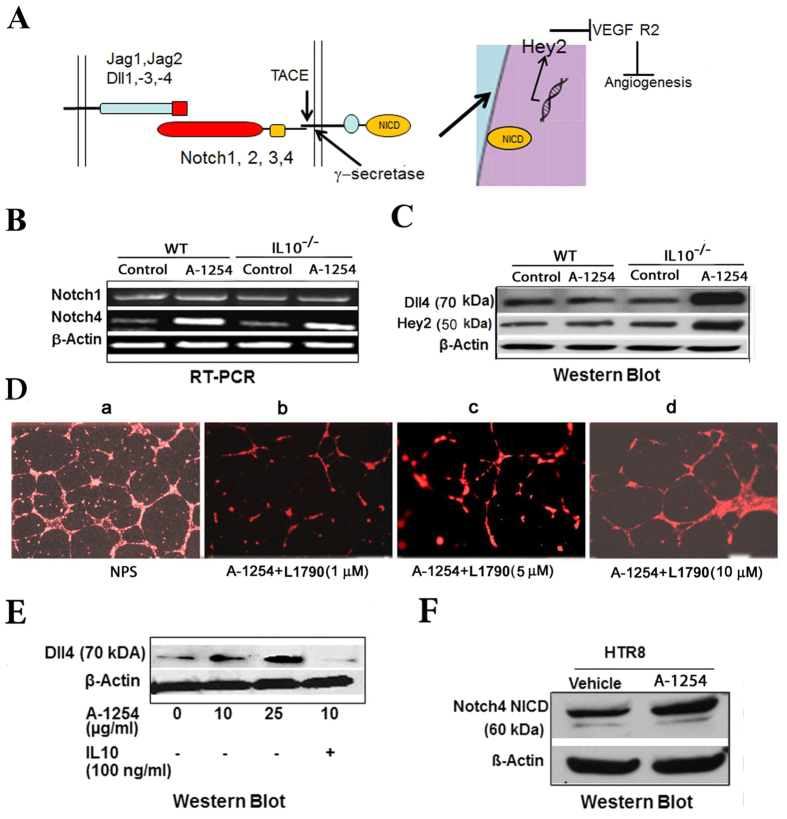
A-1254 treatment activates the Notch4/Dll4 pathway in the IL-10^−/−^ mouse placenta and human trophoblast HTR8 cells. Panel A: A schematic diagram of the interaction between Notch receptors and their ligands is shown to depict cleavage of Notch receptors by γ-secretase and translocation of Notch intracellular domain (NICD) to the nucleus. NICD activates expression of transcription factor Hey2 which in turn inhibits expression of VEGF R2. Panel B shows the effects of A-1254 treatment on the Notch1 and Nothch4 mRNA levels in the placenta of WT and IL-10^−/−^ mice treated with vehicle or A-1254 as analyzed by RT-PCR. β-Actin was used as an internal control. Panel C: Western blot analysis of Dll4 and Hey2 proteins was performed using protein extracts from the placenta obtained from WT and IL-10^−/−^ mice treated as described in panel B. Panel D shows that disruption of HUVEC-based three-dimensional tube structures by A-1254 (10 μg/ml) could be rescued by L1790, a γ-secretase inhibitor in a dose dependent manner. All the sub-panels in panel D contained NPS. Panels E and F show the results of Western blot analyses of Dll4 and Notch4 NICD in HTR8 cells after treatment with A-1254. In Panel E, IL-10 was assessed for its ability to reverse the effects of A-1254 on Dll4 activation. In Panel F, A-1254 treatment is shown to induce higher levels of Notch NICD.

Our results so far indicate that endothelial and trophoblast cells, two key cellular players at the maternal-fetal interface, may be affected by A-1254 treatment. Next, we tested whether A-1254 treatment could modulate expression of Dll, Notch, and Hey2 molecules in the placenta obtained from PCB-treated wild type (WT) or IL-10^−/−^ mice. Figure 4A presents a schematic description of the activation of Dll and Notch signaling pathways. Interaction between Notch and Dll leads to cleavage of Notch to generate Notch nuclear catalytic domain (NICD). We examined mRNA or protein levels of key Dll and Notch molecules, Hey2 transcription factor, and NICD. Figure 4B shows data on mRNA levels of Notch 1 and Notch4. A-1254 treatment did not affect the Notch1 mRNA levels in either WT and IL-10^−/−^ mouse placenta. Interestingly, Notch4 was up-regulated in the placenta of both WT and IL-10^−/−^ mice. No changes were observed in protein levels of Dll4 and Hey2 in the placenta of A-1254-treated WT mice (Fig. 4C). In contrast, A-1254 significantly increased the expression of both of these molecules in the placenta from IL-10^−/−^ mice (Fig. 4C). This indicated that IL-10 plays an important protective role in suppressing response to PCB treatment, in agreement with our previous observations^11^. Several studies have used either Notch and Dll antibodies or γ-secretase inhibitors to provide direct evidence for the involvement of Notch/Dll activation in the angiogenic pathways^51^,^52^,^53^. To confirm whether the A-1254-induced activation of the Notch/Dll pathway is responsible for anti-angiogenic activity, we assessed whether disruption of HUVEC-mediated three-dimensional tube formation could be rescued by co-treatment with L1790, a γ-secretase inhibitor. The γ-secretase inhibitors such as L1790 have been shown to suppress Notch signaling in the neonatal mouse ovary and can target the active site transmembrane cleaving aspartyl proteases^54^,^55^. Figure 4D presents a representative experiment on L1790-mediated rescue of A-1254-disrupted HUVEC-based three-dimensional structures. Our data clearly suggest that L1790 could rescue three-dimensional tube formation in a dose-dependent manner. To further confirm the protective role of IL-10, we next explored the possibility of Dll4 activation in human trophoblast HTR8 cells and its inhibition by IL-10. As expected, A-1254 activated Dll4 expression in HTR8 cells in a dose dependent manner, and IL-10 reversed this change in response to a dose of A-1254 that induced Dll4 (Fig. 4E). This is in agreement with the rescue of A-1254-induced pregnancy complications and impaired spiral artery remodeling by recombinant IL-10 *in vivo*^11^. To assess whether Dll4 induction led to cleavage of Notch4 as discussed in Fig. 4A, our data using NICD-specific antibody show significantly increased presence of Notch4 NICD in HTR8 cells treated with A-1254 (Fig. 4F).

### Exposure to A-1254 inhibits VEGF R2 in IL-10^−/−^ mouse placenta and in human trophoblast HTR8 cells or HUVECs

**Figure 5:**
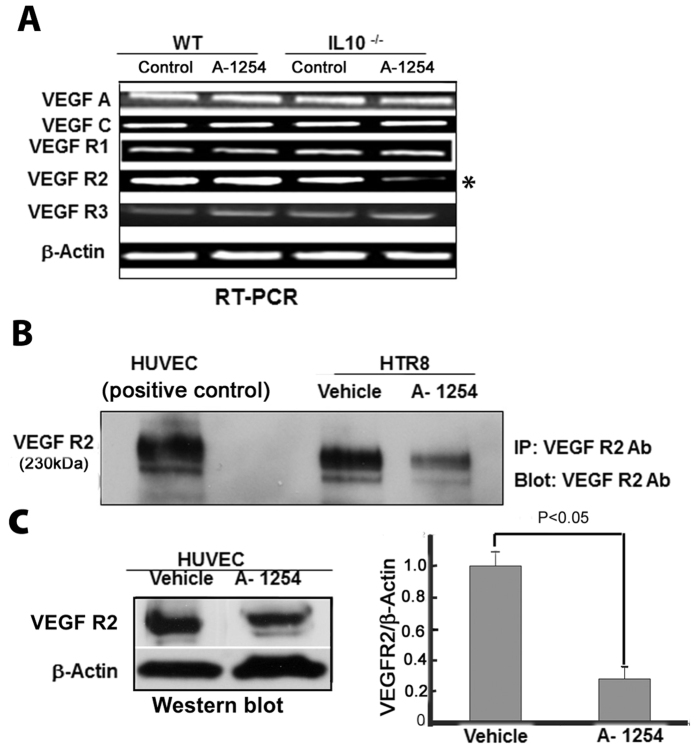
The effect of A-1254 on VEGF A and VEGF C and their receptors, VEGF R1, VEGF R2 and VEGF R3. Panel A: RT-PCR analyses were performed using total RNA from the placenta obtained from WT and IL-10^−/−^ mice treated with A-1254 or vehicle as described in Methods. The data clearly show that only VEGF R2 was inhibited as shown in the lane marked with an asterisk. β-Actin was used as an internal control for RT-PCR. Panel B: Immunoprecipitation analysis of VEGF R2 in HTR 8 cells treated with A-1254 or vehicle. Immunoprecipitates were probed for VEGF R2. Cell lysate from HUVEC cells was used as a positive control for the size identification of VEGF R2. Panel C shows a decrease (P < 0.05) in VEGF R2 protein levels in HUVECs in response to A-1254 treatment. This panel shows a cropped figure for a Western blotting experiment run under the same experimental conditions. Two extra lanes that were co-run with the lanes shown in this panel have been removed.

As discussed above, a major consequence of activation of the Notch4-Dll4-Hey2 pathway is disruption of the balance between angiogenic and anti-angiogenic activities, particularly inhibition of VEGF R2. To assess whether similar molecular mechanisms are the target of PCBs in the mouse placenta and in human trophoblasts or endothelial cells, we performed an extensive analysis of the expression of key VEGFs (VEGF A and VEGF C) and their receptors (VEGF R1, VEGF R2, and VEGF R3) in the placenta of A-1254 or vehicle treated WT and IL-10^−/−^ mice. As shown in Fig. 5A, the RT-PCR mRNA data suggest that A-1254 treatment uniquely inhibited VEGF R2 expression only in IL-10^−/−^ mice without influencing expression of other molecules in the VEGF family. To further confirm these observations, we used human trophoblast HTR8 cells. A-1254 or vehicle-treated HTR8 cells were assessed for the expression profile of VEGF R2. HUVEC cell lysates were used as positive control. Our pilot experiments suggested that HTR8 cells moderately expressed VEGF R2 as compared to HUVECs. Thus, we performed immunoprecipitation and Western blotting using HTR8 cell lysates and VEGF R2 antibody. Figure 5B shows that A-1254 significantly inhibited VEGF R2 protein content in HTR8 cells. The data presented in Figs 2 and 3A suggest that PCBs also impaired angiogenic transformation of endothelial cells. Thus, we also examined the effect of A-1254 treatment on VEGF R2 in HUVECs. The data presented in Fig. 5C suggest that A-1254 significantly inhibited VEGF R2 in HUVECs, suggesting that VEGF R2 is a novel target of PCBs in the mouse placenta as well as in human trophoblasts and endothelial cells.

## Discussion

In this study, we report for the first time that PCB molecules present in Aroclor 1254 (A-1254) mixture act as anti-angiogenic agents. Using several complementary approaches, we demonstrate that exposure to A-1254 disrupts angiogenic machinery and endovascular interaction between endothelial cells and trophoblasts. A-1254 was not found cytotoxic to either human endothelial cells or trophoblasts up to the dose of 25 μg/ml. Our data strongly suggest that human HTR8 trophoblasts treated with A-1254 failed to migrate toward or participate in cell-cell interaction with endothelial cells. A-1254 treatment also exerted its anti-angiogenic effects on a single cell level as it inhibited endothelial cell outgrowth/sprouting and capillary vessel formation in cultured mouse aortic rings. The novel findings of A-1254-mediated activation of the Notch4/Dll4/Hey2 pathway resulting in inhibition of VEGF R2 expression in the mouse placenta and human trophoblast or endothelial cells may explain placental insufficiency and pregnancy disruption caused by PCBs.

Our published observations showed that exposure to A-1254 caused preterm birth, fetal growth restriction, and amniotic fluid increase in IL-10^−/−^ mice^11^. Importantly, A-1254 treatment during pregnancy induced neurological anomalies in the offspring. Since these observations were not observed in WT mice irrespective of acute doses used, it was demonstrated that IL-10 was protective against PCB exposure, albeit during the gestation period in mice. IL-10 is a potent anti-inflammatory and vascular cytokine^26^,^27^. Absence of IL-10 may predispose to inflammatory activities as well as poor vascularization at the maternal-fetal interface. The data presented in Figs 4 and 5 clearly suggest that IL-10 could reverse the effect of PCBs on Dll4 induction and inhibition of VEGF R2.

As discussed above, we used a high dose of A-1254 in pregnant mice for a short period of time to induce acute effects. Comparing the PCB levels in environment and in experimental settings is a complex task. First, in the experimental settings, doses are chosen to see effects in a very short period of time. Second, A-1254 is a mixture of more than 100 PCB congeners and only a few of them will have pregnancy-disrupting activity. In this regard, our preliminary data suggest that a single coplanar congener, PCB126, can mimic the effects of A-1254 at a 500 fold lower dose. Humans are generally exposed via the ingestion of food containing small amounts of PCBs, but in certain circumstances humans may be exposed via inhalation. Human serum levels in highly exposed individuals, such as those residing around the production facility in Anniston, Alabama, show median levels of 528 ng/g lipid, with the highest reported in excess of 27 μg/g lipid^56^. Exposure to PCBs continues since PCBs are produced as byproducts in pigment and dye manufacture, resulting in printed materials and paints contaminated with them. These new sources of PCBs along with the continuing use of products produced before the ban, especially building materials, represent sources of continuing exposure^57^. It is possible that differences in how individuals detoxify and eliminate PCBs from the body may explain the disparity. Genetic susceptibility may be another important factor as shown in our study.

VEGFs mediate their intracellular activity by binding and signaling through their cognate VEGF receptors^36^. In the case of human trophoblasts, we have demonstrated that VEGF receptors, particularly VEGF R2, are abundantly expressed on first trimester trophoblasts compared to third trimester trophoblasts^33^. Significantly, VEGF R2, which mediates intracellular signaling, was found to be down-regulated by A-1254 treatment in IL-10^−/−^ placenta without any significant effect in wild type mice (Fig. 5). The reduced expression of VEGF R2 was also seen at the protein level in human HTR8 trophoblasts and HUVECs (Fig. 5). Using a number of complementary approaches, we have provided evidence for A-1254-mediated inhibition of angiogenic activity (Figs 1, 2, 3). Since VEGF R2 was found to be a critical target of PCB treatment and is a key regulator of angiogenesis, the attenuated response to angiogenic signals observed as a result of A-1254 treatment could be due to reduced expression of VEGF R2.

A major pathway that controls regulation of VEGF R2 is the Notch/Dll signaling pathway. Notch/DLL activation has been associated with both pro-angiogenesis and anti-angiogenic signaling^39^,^46^,^47^,^49^,^58^,^59^,^60^,^61^,^62^,^63^. In the case of the human and mouse placenta, endovascular trophoblasts have been shown to exhibit highest Notch activity^39^. Thus, it is tempting to speculate that microenvironment coupled with temporal need and regulation of the activation of Notch/Dll pathway may guide whether this pathway is pro-angiogenic or anti-angiogenic. Our data demonstrate that A-1254 treatment induced the expression of Dll4 and its cognate receptor Notch 4 in the IL-10^−/−^ placenta (Fig. 4). Consistent with Dll4 signaling through Notch, expression of HEY2, one of the transcription factors that mediates Notch/Dll functions, was significantly induced in the IL-10^−/−^ placenta. Hey 2 is a basic helix loop helix (bHLH) transcription factor that has been reported to down-regulate VEGF R2 expression by inhibiting its promoter activity^46^. Our data from A-1254-treated mouse placenta or human trophoblast and endothelial cells are in agreement with these observations. We also provide evidence that suppression of Notch signaling by L1790, a γ-secretase inhibitor, could rescue angiogenic transformation of HUVECs (Fig. 4D). To best of our knowledge this is the first report on the Dll4-Notch4-VEGFR2 axis as a target of PCBs. Our studies are likely to provide a basis for congener-specific studies to delineate the role of coplanar and non-coplanar PCBs in angiogenesis and inflammation.

## Conclusions

Overall, our study suggests that the Dll4-Notch4 signaling pathway is a novel target of PCBs during gestational exposure, particularly when coupled with genetic stress like IL-10 deficiency. This study further highlights that multiple cell types would be influenced by PCBs at the maternal-fetal interface resulting in poor spiral artery remodeling and attenuated placental angiogenesis. Thus, contribution of environmental toxicants such as PCBs to the increasing incidence of pregnancy complications cannot be underestimated.

## Methods

### Animals

All animal protocols were approved by the Lifespan Institutional Animal Care and Use Committee and carried out in accordance with the relevant guidelines. Mice were housed and mated in a specific pathogen-free facility under the care of the Central Research Department of Rhode Island Hospital. All mating experiments were repeated at least three times with at least three mice per treatment. The day of vaginal plug appearance was designated gestational day (gd) 0. We administered daily i.p injection of 500 μg A-1254 (Sigma-Aldrich) per mouse or an equivalent volume (100 μl) of vehicle (corn oil) to pregnant C57BL/6 wild type mice or their IL-10^−/−^ counterparts from gd 4 to 12. Uteroplacental units were collected on gd 13 and used for further analysis as described^11^.

### Cell culture and treatment

Immortalized first trimester trophoblast cell line HTR8 with properties of invasive extravillous cytotrophoblasts was established and kindly provided by Dr. Charles Graham^64^. HTR8 cells were grown to ~80% confluence in RPMI standard growth medium and used only during eight passages. Primary human umbilical cord endothelial cells (HUVEC) were obtained from Cambrex and cultured in EBM-2 medium (Cambrex). All cells were maintained in standard culture conditions of 5% CO_2_ at 37 °C. All experiments utilized cells at passage six or less.

### *In vivo* matrigel plug assay

*In vivo* Matrigel plug assay was performed as described^34^. Matrigel was obtained from BD Biosciences. Briefly, a 600 μl mixture of HUVECs (2 × 10^5^) and growth factor reduced-Matrigel resuspended in 10% normal pregnancy serum (NPS) was injected subcutaneously (s.c.) into nude mice that resulted in the formation of palpable plugs. Thirty minutes later, 7.5 × 10^5^ trophoblast cells (HTR8) treated for 24 hr with A-1254 (10 μg/ml) or vehicle (DMSO) were injected at the rim of the Matrigel plug. The Matrigel plugs were excised 6 days post injection and the cells were isolated using cell recovery solution (BD Biosciences) and stained with a monoclonal antibody against human cytokeratin 7 (CK7) (mouse anti-cytokeratin-7, clone RCK105, BD Biosciences) and FITCH-conjugated anti-mouse IgG1 (eBioscience, #11–4015–80 anti-mouse IgG1 FITC) and quantified FACS analysis using FACSCanto (Becton Dickinson).

### Aortic ring assay

Aortic ring assays were performed to assess the ability of A-1254 to inhibit endothelial cell sprouting and formation of micro-vessels. Briefly, aortic arches were removed from euthanized mice and immediately transferred to a culture dish containing ice-cold serum-free media. Aortic rings (1 mm thick) were sectioned and extensively rinsed in 5 consecutive washes of Medium 200 (Gibco). Ring-shaped explants of aorta were then embedded in Matrigel- coated 48 well plates, treated with A-1254 at varying doses (0, 1, 10, 25 μg/ml) in the presence or absence of serum and incubated at 37 °C in a tissue culture incubator. Growth medium with A-1254 was replaced every 48 hrs. On the sixth day, the endothelial cell outgrowth/sprouting from aortic ring explants were monitored and recorded using Nikon Eclipse TS100 inverted microscope. The sprouting of micro-vessels from aortic rings in the Matrigel was quantified by using the MTS assay as described previously^64^.

### Endothelial capillary tube formation assay

Briefly, growth factor-depleted Matrigel was thawed overnight at 40 °C and mixed to homogeneity. Culture plates (48-well) were coated with 0.1 ml of Matrigel and allowed to gelatinize at 37 °C for 30 min. HUVECs or HUVECS and HTR8 cells (2.5 × 10^4^ each), labeled with cell tracker Red CMTPX and Green CMFDA (Molecular Probes), respectively, were cultured on Matrigel in the presence of 10% normal pregnancy serum (NPS) containing A-1254 (10 μg/ml). The concentration of A-1254 was selected from a dose response studies described earlier^11^. The endothelial cell tube formation was monitored and recorded after 12–14 hrs of incubation using the florescence microscopy at 4 x magnification (Nikon Eclipse TS 100 coupled with CCD camera) as described^11^,^33^. To evaluate the significance of the Notch/Dll pathway in A-1254-induced inhibition of angiogenesis, A-1254-induced disruption of three-dimensional tube formation by HUVECs was assessed in the presence or absence of different doses of γ-secretase inhibitor, L1790 (0, 1, 5, 10, 25 μM) (Sigma-Aldrich).

### Cell viability assay

The effect of A-1254 on cell viability of first trimester (HTR-8 and 3 A) and third trimester (TCL-1) trophoblast cell lines was determined by the Cell Titre 96 AQueous One Solution Proliferation Assay’ kit (Promega) following the manufacturer’s recommendations with suitable modifications by the first author^33^. Briefly, trophoblast cells (2 × 10^4^ cells) were plated in a 48-well plate, and allowed to attach overnight. Medium was replaced with fresh complete medium containing various concentrations of A-1254. Stock solutions of A-1254 were prepared in DMSO, serially diluted in complete media, added to the wells and incubated at 37 °C for required periods (72 hours) in a cell culture incubator. Appropriate vehicle controls were included in all experiments. MTS reagent was added, incubated for further 2 hours to develop color and absorbance was measured at 490 nm using a microplate reader (Titertek Multiscan Microplate Reader, Diversified Equipment). In this assay, the absorbance is directly proportional to the number of living cells and hence is a useful screening assay for cytotoxicity. Cell viability is represented as the mean percentage ±SD of absorbance.

### Semi-quantitative RT-PCR

Total RNA was isolated from gd 13 placenta according to the manufacture’s protocol by using RNeasy Kit (QIAGEN). 2 μg of RNA was used for the first strand cDNA synthesis by Superscript III (Invitrogen). The primer sets for mouse VEGF A, VEGF C, VEGF R1, VEGF R2, VEGF R3, Notch 1, Notch 4, and β actin were as follows: VEGF A, 5′-CAC CGG GTT GGG TTG TCA CAT -3′ (F) and 5′-CAC CGC CTT GGC TTG TCA CAT-3′ (R), (291 bp product); VEGF C, 5′-TGA ACA CCA GCA CAG GTT AC-3′ (F) and 5′-TCT TGT TAG CTG CCT GAC AC-3′ (R) (360 bp); VEGF R1, 5′-GCG CAT GAC GGT CAT AGA AGG A-3′ (F) and 5′-TGA GGT TTT GAA GCA GGT GTG G-3′ (R), (481 bp); VEGF R2, 5′-ATC CAA GCT GCC AAC GTC TCA GC-3′ (F) and 5′-TGG GTG CCA TGC GCT CTA GGA T-3′ (R) (447 bp); VEGF R3, 5′-CAG ACA GAC AGC GGG ATG GTG C-3′ (F) and 5′-AGG CTG TAG TGG GGG TGG GAC A-3′ (R) (410 bp); Notch 4, 5′-CAG CCC GAG CAG ATG TAG GA-3′ (F) and 5′-CGG CGT CTG TTC CCT ACT GT-3′ (R), (145 bp); Notch 1, 5′-GTC AAT GCC GTG GAT GAC CT-3′ (F), 5′-TCA CAC TGG CCA TTC AAG CT-3′ (R) (865 bp); and β actin, 5′-TCC TTT GCA GCT CCT TGG TTG CCG -3′ (F) and 5′-TGG ATG GCT ACG TAC ATG GCT GGG-3′ (R) (457 bp). All PCR reactions were conducted using GenAmp 9600 PCR system (Perkin-Elmer). The RT-PCR conditions were standardized for each product with varying cycles of reaction.

### Immunoprecipitaion and Western blotting

Cells were lysed in RIPA buffer (50 mM Tris-HCl (pH 7.4), 1% Nonidet P-40, 0.25% sodium deoxycholote, 150 mM NaCl, 1 mM EDTA, 1 mM NaF, and proteinase inhibitor cocktail). A total of 200 μg of cell lysate and 10 μl rabbit monoclonal anti-VEGFR-2 antibody (no.2749, Cell Signaling Technology; 1:100 dilution) were incubated in a shaker at 4 °C overnight. After 2 h of incubation with 25 μl of protein A/G ultraLink Resin (Thermo Scientific) at 4 °C, the beads were washed three times with PBS then boiled in SDS-PAGE sample buffer for 5 min to elute proteins for subsequent Western blotting for VEGF R2. For Western blotting, the proteins from placental tissue or first trimester HTR8 trophoblast cell lysates were separated on 12% SDS–polyacrylamide gels and blotted onto PVDF membranes and probed with anti-mouse antibodies for VEGF A, VEGF C, Dll4 (Abcam), Hey 2 and β-actin (Biovision). We used ECL chemiluminescence (Amersham Biosciences) to visualize the bands and recorded them using Konica SRX 101 A developer.

### Statistical analysis

We performed statistical analysis using one way ANOVA and two tailed Student’s‘t’ test using Data Analysis tool in Microsoft Excel. Statistical significance was set at P < 0.05.

## Additional Information

**How to cite this article**: Kalkunte, S. *et al*. Polychlorinated biphenyls target Notch/Dll and VEGF R2 in the mouse placenta and human trophoblast cell lines for their anti-angiogenic effects. *Sci. Rep.* **7**, 39885; doi: 10.1038/srep39885 (2017).

**Publisher's note:** Springer Nature remains neutral with regard to jurisdictional claims in published maps and institutional affiliations.
